# Transforming capacity-strengthening in an era of sustainable development

**DOI:** 10.1007/s00038-020-01490-4

**Published:** 2020-10-22

**Authors:** Anne Christine Stender Heerdegen

**Affiliations:** 1 Swiss Tropical and Public Health Institute, Basel, Switzerland; 2 University of Basel, Basel, Switzerland

District health managers (DHMs) operate and manage most public health care facilities in the health systems of many low- and middle-income countries (LMICs). They must turn national health policies and human, material, and financial resources into accessible high-quality health services (Heerdegen et al. 2020a). To improve district health services in many LMICs, the capacity of DHMs must be strengthened (Dovlo 2016).

Capacity-strengthening is multi-dimensional and includes “*efforts towards strengthening the ability of individuals, organizations, or institutions to perform appropriate functions effectively, efficiently, and sustainably*” (Bates et al. 2011). Many district management strengthening interventions focus on strengthening the DHMs’ individual competency through formal learning, e.g., classroom training (Martineau et al. 2018). Improving the skills, knowledge, and attitudes of DHMs increases their ability to carry out their job, but their performance also depends on the environment in which they operate (Heerdegen et al. 2020b). In Ghana, for example, DHMs face resource uncertainty (human, material and financial), have limited decision-making authority, and operate under budgetary restrictions in challenging working conditions (Heerdegen et al. 2020b). No matter how competent the DHMs are, these factors affect their ability to carry out responsibilities and limits their opportunity to be innovative and agile in responding to district health needs (Heerdegen et al. 2020b).

Capacity should be viewed systemically because it is a product of a complex combination of factors (i.e., individual competencies, resources, policies) in a specific context (Aragón and Giles Macedo 2010). Efforts to strengthen capacity must be based on a comprehensive understanding of the context in which DHMs are embedded. Gaining this understanding requires actively involving local stakeholders, including DHMs, who can identify bottlenecks and inefficiencies in organizational processes, policies, or individual competencies (de Savigny and Adam 2009). They can provide expert knowledge about management capacity gaps, and suggest ways to fill these sustainably, in ways appropriate to their culture and context.

Actively involving local stakeholders in a participatory approach to capacity-strengthening is made easier by the use of tools that encourage users to think thematically, like social network analyses, causal loop diagrams, problem trees, and process mapping (de Savigny and Adam 2009). Process mapping has for example helped in effectively identifying inefficiencies in health workforce managerial practices in Ghana (Heerdegen et al. 2019). Actively involving local stakeholders can also make it easier for implementers of management strengthening efforts to identify existing national, regional, and local strategies, policies, and plans that contribute to strengthen management capacity.

Enhancing *“international support for implementing effective and targeted capacity-building in developing countries to support national plans to achieve the SDGs”* is a Sustainable Development Goal (SDG 17: ‘Partnerships for Sustainable Development’), but bilateral development partners who are responsible for capacity-building initiatives in LMICs often disregard country policies and plans (United Nations 2019). This is problematic as capacity-building efforts are less sustainable when not integrated into the national health system (Bates et al. 2011). Moreover, it may create perpetual dependency on development partners (Brinkerhoff 2007). Development partners may bypass national plans to strengthen capacity because they are unaware of them—a situation that underscores the importance of collaborating closely with local stakeholders.

A systemic and participatory approach to strengthening management capacity may be more time-consuming than traditional competency development interventions, as it requires building relationships and a level of trust that facilitates effective partnerships with local stakeholders. Moreover, contextual and organizational factors like policies, structures, and resource availability that undermine a DHM’s ability and willingness to carry out their duties are more complex to address compared with individual competencies (Brinkerhoff 2007). Nonetheless, without collaborating with local stakeholders, interventions are unlikely to be sustainable, and if the system in which the DHM is embedded does not change, many competent DHMs will still have limited capacity to carry out their responsibilities effectively and efficiently. In turn, this may limit the public’s access to essential and responsive health services at the district level.

Targeting competencies alone is insufficient to develop sustained improvements in management capacity. Since DHMs’ capacity is determined by a complex interaction of individual, organizational, and contextual factors, future capacity-strengthening initiatives should take on a systemic approach that utilizes tools to encourage systemic thinking, and encourages close collaboration with relevant local stakeholders (Heerdegen et al. 2020b).
